# Efficient simulation of cardiac electrical propagation using high order finite elements

**DOI:** 10.1016/j.jcp.2012.01.037

**Published:** 2012-05-20

**Authors:** Christopher J. Arthurs, Martin J. Bishop, David Kay

**Affiliations:** a Department of Computer Science, University of Oxford, Oxford, United Kingdom; b Department of Biomedical Engineering, King’s College London, London, United Kingdom

**Keywords:** Finite element method, *p*-Version, Monodomain simulation, Computational cardiology, Numerical efficiency

## Abstract

We present an application of high order hierarchical finite elements for the efficient approximation of solutions to the cardiac monodomain problem. We detail the hurdles which must be overcome in order to achieve theoretically-optimal errors in the approximations generated, including the choice of method for approximating the solution to the cardiac cell model component. We place our work on a solid theoretical foundation and show that it can greatly improve the accuracy in the approximation which can be achieved in a given amount of processor time. Our results demonstrate superior accuracy over linear finite elements at a cheaper computational cost and thus indicate the potential indispensability of our approach for large-scale cardiac simulation.

## 1 Introduction

New insight into cardiac function is of great importance to medical science, not least because heart disease is the leading cause of death in the developed world; in the United Kingdom alone it accounts for more than one in six of all deaths [1]. Increased understanding of the working of the heart in both physiological and pathological conditions will therefore aid the development of new treatments for a variety of cardiac and non-cardiac [2,3] diseases.

A major hurdle we face is that obtaining high spatial and temporal resolution data on the dynamics of the heart is difficult. In a clinical environment, we must settle for low resolution methods such as magnetic resonance imaging (MRI) or electrocardiograms (ECG). Outside of the clinical setting, more information can be obtained from animal studies, for example by optical mapping [4]. Unfortunately, these only provide incomplete data on a limited subset of the parameters of interest. Computational multiscale simulation provides another tool, allowing the measurement and modification of hundreds of different variables in the whole three-dimensional tissue volume, with the added benefits of procedural simplicity and avoiding the need for animal studies.

Myocardial electrical propagation can be simulated using the monodomain or bidomain PDEs [5,6]. Due to its capacity to represent complex geometries with ease, approximations are often obtained using the finite element method (FEM) to discretise the PDEs in space on realistic cardiac geometry meshes; this results in very large (up to forty-million degrees of freedom (DOF) for human heart geometries) systems of linear equations which must be solved many thousands of times over the course of even a short simulation. Thus, they are extremely computationally demanding, presenting taxing problems even to high-end supercomputing resources. This computational demand means that effort has been invested in developing efficient solution techniques, including work on preconditioning, parallelisation and adaptivity in space and time [7–12]. In this study, we investigate the potential of reducing the number of DOF by using a high-order polynomial FEM [13–15] to approximate the monodomain PDE in space, with the goal of significantly improving simulation efficiency over the piecewise-linear FEM approach commonly used in the field [16–19]. For schemes where the polynomial degree *p* of the elements is adjusted according to the error in the approximation, this is known as the finite element *p*-version. In the work presented here, we work with schemes which keep *p* fixed.

Because the *a priori L*^2^ error ∥*u* − *u*_*hp*_∥_0,2_ between the true solution *u* and finite element approximation *u*_*hp*_ of spatially semi-discrete parabolic PDEs satisfies

(1) $$\|u-u_{\mathrm{hp}}{\|}_{0\text{,}2}⩽{\mathrm{Ch}}^{\mu }p^{-k}\|u{\|}_{k\text{,}2}\text{,}$$

on a mesh with quasiuniform element diameter *h*, for some constant *C* with *μ* = *min{k*, *p* + 1} (when the true solution has *k* square-integrable derivatives, allowing the norm ∥ · ∥_*k*,2_, which we recall later, to exist) [15], and because for our problem we believe that *k* is large, we have good reason to look for greater computational efficiency from using larger values of *p* and *h* in order to obtain a desired accuracy. This allows for an exponential rate of error reduction as *p* increases. It is reasonable that we should expect *k* to be large given that our problem is a homogenised model of a physical process. Careful choice of *h* and *p* can result in a linear system with fewer DOF and thus improved computational efficiency. In other fields such as acoustics, elastodynamics and electromagnetics, this approach has been shown to produce a speed-up of five to ten times over a standard linear FEM approach [20].

Work to-date on higher-order elements [21,22] has focused on hexahedral meshes and what is effectively lumping of the mass matrix [23] (despite claims that an advantage of such high-order methods is that they avoid mass-lumping [20]). The existing approaches demonstrate a two- to threefold speed up over linear FEM for a 3D parabolic test problem on a coarse cardiac geometry [22]. Our approach allows the use of tetrahedral meshes and lends itself to spatial adaptivity, although we do not investigate the latter here.

In this study, we focus on the monodomain equation, although the presented techniques can easily be extended to the bidomain problem. In one spatial dimension, we provide comparisons of the error in the simulations using different polynomial degrees with theoretical *a priori* results in certain norms, displaying strong agreement. We also use simpler error measures such as activation times and conduction velocities (CV); these are applied in both the one-and two-dimensional cases.

## 2 Methods

### 2.1 Introducing the governing equations

The myocardium consists of roughly cylindrical cardiac myocytes which are connected to their neighbours by gap junctions, creating an electrically-connected syncytium known as the intracellular space which sits within the extracellular space. The myocytes are arranged into thin fibres, aligned axially. These fibres are in turn arranged into sheets of myocardial tissue. The coordinated contraction of the myocytes which makes the heart beat is orchestrated by spatial waves in the electric potential difference between the intracellular and extracellular spaces, which we refer to as the transmembrane potential. These waves are caused by triggered pulses in the transmembrane potential on a cellular level, called action potentials (APs), triggering further APs in neighbouring cells via tissue-level electrical conductivity.

For isolated cells, APs can be simulated using a system of ODEs [24–27], or [28] for a detailed exposition, describing how the cellular machinery of the myocytes controls the flux of ionic species through ion channels across the cell membrane. Full cardiac tissue simulation is made tractable via a homogenisation procedure which does away with the individual cells, modelling the tissue instead as two compartments representing the intracellular and extracellular spaces; both are considered to exist at every point in the domain. The spaces are electrically-isolated except for the transmembrane ionic currents between them, which are given by PDE analogues of the isolated cell ODE models. This homogenisation results in the bidomain reaction–diffusion-type system of differential equations describing AP propagation. It consists of two PDEs describing the electrical conduction in the intracellular and extracellular spaces coupled to the PDE system for the transmembrane ionic currents and concentrations *w*. We must be careful how we discretise the latter, as we shall demonstrate. The two spaces display anisotropic conductivity due to the sheets and fibres; in order to capture this, each has an associated conductivity tensor. Approximating these as being proportional to one another, this system can be reduced to the monodomain: a single PDE describing the dynamics of the transmembrane potential together with a formulation for *w* that remains unchanged from the bidomain. For more detail, see for example [6,29].

The results obtained with the monodomain equation will not be identical to those found with the bidomain, differing in CV by around 2% [30], but because of the difference in computational effort required to solve the two forms, the researchers use the monodomain where possible. Monodomain simulations can reproduce most of the behaviour seen when using the bidomain, including some which involve phenomena which once required the bidomain such as bath-loading effects [31], but excluding defibrillation due to the need to simulate virtual electrodes [32]. The techniques that we present here are expected to extend without difficulty to the bidomain.

The monodomain system with a cell model is given by

(2a) $$C_{m}\frac{\partial u}{\partial t}-\frac{1}{\beta }\nabla \cdot (\sigma \nabla u)-I_{\mathrm{ionic}}(u\text{,}w)=I_{\mathrm{stim}}(x\text{,}t)\;\text{in}\;\Omega \text{,}$$

(2b) $$\frac{\partial w}{\partial t}-g(u\text{,}w)=0\;\text{in}\;\Omega \text{,}$$

(2c) $$u(x\text{,}0)=u_{0}(x)\;\forall x\in \Omega \text{,}$$

(2d) $$\overset{ˆ}{n}\cdot (\sigma \nabla u)=0\;\text{on}\;\partial \Omega \text{,}$$

(2e) $$w(x\text{,}0)=w_{0}(x)\;\;\forall x\in \Omega \text{,}$$

where *x* is a point in the *n*-dimensional myocardial domain *Ω*, *Ω* has boundary *∂Ω* which is often polygonal due to the methods used to generate it from cardiac MRI [33], outward-pointing unit surface normal $\overset{ˆ}{n}$ to *∂Ω*, transmembrane potential *u*(*x*, *t*), initial conditions *u*_0_ and *w*_0_, conductivity tensor *σ*(*x*) (related to fibre orientation), time *t*, cell membrane capacitance *C*_*m*_, surface area to volume ratio *β* and current *I*_*total*_(*u*, *w*, *x*, *t*) = *I*_*ionic*_(*u*, *w*) + *I*_*stim*_(*x*, *t*), consisting of *I*_*ionic*_(*u*, *w*) the transmembrane ionic current as described by the cell model and *I*_*stim*_(*x*, *t*), the stimulus current as determined by the experimental protocol. *g* describes how the *m* cell model state variables *w*(*x*, *t*) = (*w*_1_(*x*, *t*), … , *w*_*m*_(*x*, *t*))^*T*^ vary in time. In this work, we use the Luo–Rudy Phase I (LR91) cell model [24], modified according to [34], for *g* and *I*_*ionic*_. We are primarily interested in the transmembrane potential *u*.

### 2.2 Discretisation

For the space discretisation of the transmembrane potential PDE, the FEM approach is outlined in what follows. We recall the definition of the Hilbert space $H^{1}(\Omega )$,

$$H^{1}(\Omega )≔\{\chi :\Omega \to R|\|\chi {\|}_{1\text{,}2}<\infty \}\text{,}$$

where

$$\|\chi {\|}_{m\text{,}q}={\left(\underset{0⩽|\alpha |⩽m}{\sum }\int_{\Omega }|D^{\alpha }\chi {|}^{q}\mathrm{dx}\right)}^{\frac{1}{q}}\text{,}$$

with $\alpha =(\alpha_{1}\text{,}\ldots \text{,}\alpha_{n})\text{,}|\alpha |=\sum_{i=1}^{n}\alpha_{i}$ and $D^{\alpha }≔D_{1}^{\alpha_{1}}\ldots D_{n}^{\alpha_{n}}$, where $D_{i}≔\frac{\partial }{\partial x_{i}}$ [35]. In what follows, for convenience we shall often write $(a\text{,}b)=\int_{\Omega }a\cdot \mathrm{bdx}$.

We cast the transmembrane potential PDE from system (2) into its weak form: find $u\in H^{1}(\Omega )$ such that for all $\chi \in H^{1}(\Omega )$,

$$\left(u_{t}\text{,}\chi )-\frac{1}{\beta C_{m}}(\nabla \cdot (\sigma \nabla u)\text{,}\chi )=\frac{1}{C_{m}}(I_{\mathrm{total}}\text{,}\chi \right)$$

and integrate by parts, giving

$$(u_{t}\text{,}\chi )-\frac{1}{\beta C_{m}}\left(\int_{\partial \Omega }\chi \sigma \nabla u\cdot \overset{ˆ}{n}\mathrm{dx}-(\sigma \nabla u\text{,}\nabla \chi )\right)=\frac{1}{C_{m}}(I_{\mathrm{total}}\text{,}\chi )\;\forall \chi \in H^{1}(\Omega )\text{,}$$

where $\overset{ˆ}{n}$ is an outward-pointing unit surface normal. The boundary condition (2d) means that the integral on *∂Ω* is zero, so

$$(u_{t}\text{,}\chi )+\frac{1}{\beta C_{m}}(\sigma \nabla u\text{,}\nabla \chi )=\frac{1}{C_{m}}(I_{\mathrm{total}}\text{,}\chi )\;\forall \chi \in H^{1}(\Omega )\text{.}$$

Choosing some finite-dimensional subspace $S\subset H^{1}(\Omega )$ to work in, with basis ${\left\{\phi_{i}(x)\right\}}_{i=1}^{N}$, we can find a spatially discrete approximation $u_{d}=\sum_{i=1}^{N}u_{i}\phi_{i}$ to *u* by determining appropriate basis function weights *u*_*i*_. Inserting this, we obtain the semi-discrete system of equations

$$\left(\overset{N}{\underset{i=1}{\sum }}u_{t\text{,}i}\phi_{i}\text{,}\phi_{j}\right)+\frac{1}{\beta C_{m}}\left(\sigma \nabla \left(\overset{N}{\underset{i=1}{\sum }}u_{i}\phi_{i}\right)\text{,}\nabla \phi_{j}\right)=\frac{1}{C_{m}}(I_{\mathrm{total}}\text{,}\phi_{j})\;j=1\text{,}2\text{,}\ldots \text{,}N\text{.}$$

We now write ***u*** = (*u*_1_, *u*_2_, … , *u*_*N*_)^*T*^, ***I*** = ((*I*_*total*_, *ϕ*_1_), (*I*_*total*_, *ϕ*_2_), … , (*I*_*total*_, *ϕ*_*N*_)), and ***M*** and ***A*** for the matrices with (*j*, *i*)th entry (*ϕ*_*i*_, *ϕ*_*j*_) and (*σ*∇*ϕ*_*i*_, ∇*ϕ*_*j*_), the mass and stiffness matrices respectively, to obtain

(3) $${\mathrm{Mu}}_{t}+\frac{1}{\beta C_{m}}\mathrm{Au}=\frac{1}{C_{m}}I$$

which we want to solve for ***u***. More details can be found for example in [36,37].

Practically, we require a mesh $M=\{\tau_{i}\}$ of elements *τ*_*i*_ with the property that ∪_*i*_*τ*_*i*_ = *Ω* and such that when *i* ≠ *j*, if *τ*_*i*_ ∩ *τ*_*j*_ is non-empty, it is an element vertex (mesh node) or an entire edge of both *τ*_*i*_ and *τ*_*j*_. In 1D, M consists of non-overlapping line segments. In 2D, we use triangular elements. Let *h*_*i*_ = *diam*(*τ*_*i*_) ≔ sup{*d*(*x*, *y*)∣*x*, *y* ∈ *τ*_*i*_}, where *d*(·, ·) is the standard Euclidean metric on $R^{2}$, be the diameter of $\tau_{i}\text{,}\;h=\max_{i}\{h_{i}\}\text{,}\;\rho_{\tau_{i}}=\sup\{\mathrm{diam}(B)|B\text{a ball in}\tau_{i}\}$, and *Q* and *R* be constants independent of M. We work with meshes that satisfy for all *i*

(4a) $$\frac{h}{h_{i}}⩽Q\text{,}$$

(4b) $$\frac{h_{i}}{\rho_{\tau_{i}}}⩽R\text{,}$$

where relation (4a) is the property of quasiuniformity of the mesh [38].

The subspace *S* is determined in our case by the choosing M and a basis ${\left\{\phi_{i}\right\}}_{i=1}^{N}$ of continuous piecewise polynomials of degree at most *p* in each element; we denote by *S*_*hp*_ the particular *S* associated with M and *p*. The usual choice in the field is to use linear elements (*p* = 1) with *ϕ*_*i*_(*x*_*j*_) = *δ*_*i*,*j*_ ∀ *i*, *j*, where ${\left\{x_{j}\right\}}_{j=1}^{N}$ is the set of nodes of the mesh and *δ* is the Kronecker delta function. In this work we demonstrate how to work with *p* > 1 for system (2). The spaces we use can be obtained from the standard linear FEM *S*_*hp*_ = *S*_*h*1_ by hierarchically adding basis functions of increasing degree to each element. Because each basis function has a DOF associated with it, increasing *p* results in an enlargement of the system of linear Eqs. (3). However, the significantly improved accuracy that increased *p* provides allows us to more than offset this by using a coarser mesh.

**Fig. 1:**
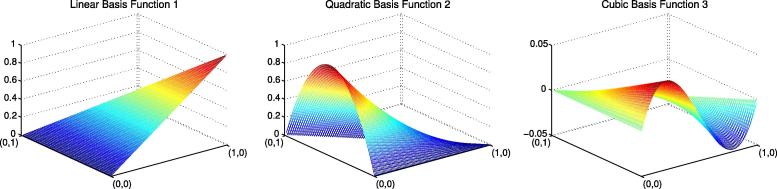
Three of the ten hierarchical basis function pieces required in 2D on the reference element for a cubic finite element approximation of the solution. Note that when mapped to the real mesh from the reference element, each of these is just part of one of the basis functions that is actually supported on multiple elements.

We take the hierarchical approach because it has the advantage that on each element the basis of degree *p* is the same as the basis of degree *p* + 1 with the degree *p* + 1 functions removed. This lends itself to adaptive techniques, although we do not explore them here. For example, with *p* = 3 on a 1D reference element [0, 1] we have

$$x\text{,}1-x\text{,}x(1-x)\;\text{and}\;x(1-x)\left(\frac{1}{2}-x\right)$$

which are the two components of a linear basis function, the quadratic and the cubic respectively. In 2D the analogous approach has three linear, three quadratic and four cubic basis functions partially or wholly supported on each element; see Fig. 1 for some of these.

### 2.3 Treating *I*_*total*_ and *w*

We wish to have an approximation to *I*_*total*_ of the form $\sum_{i=1}^{N}I_{i}\phi_{i}\text{,}I_{i}\in R\text{,}$ so that the integration required to generate ***I*** in the linear system (3) can be reduced to the computationally efficient product ***M***(*I*_1_, … , *I*_*N*_)^*T*^, referred to as matrix-based assembly. Because the term *I*_*ionic*_ in (2a) is nonlinear, in general *I*_*total*_ ∉ *S*_*hp*_. Thus we require a choice of a suitable projection $\Pi :L^{2}(\Omega )\to S_{h\overset{˜}{p}}\subset S_{\mathrm{hp}}$ for some $\overset{˜}{p}$; we can then work with *ΠI*_*total*_. Additionally, the non-diffusing cell state variables *w* must be solved for with sufficient spatial accuracy; a piecewise-linear approach to *w*, which is effectively what is used in most implementations, will limit the overall accuracy of the scheme. We can obtain the accuracy needed by using finite elements of degree $\overset{˜}{p}$ to approximate *w*; in this case there is a very natural way to construct *Π* mapping into $S_{h\overset{˜}{p}}$ (see Section 2.4), so we take the same $\overset{˜}{p}$ for the degree of the image of *Π* and the order of the finite elements used for *w*.

**Fig. 2:**
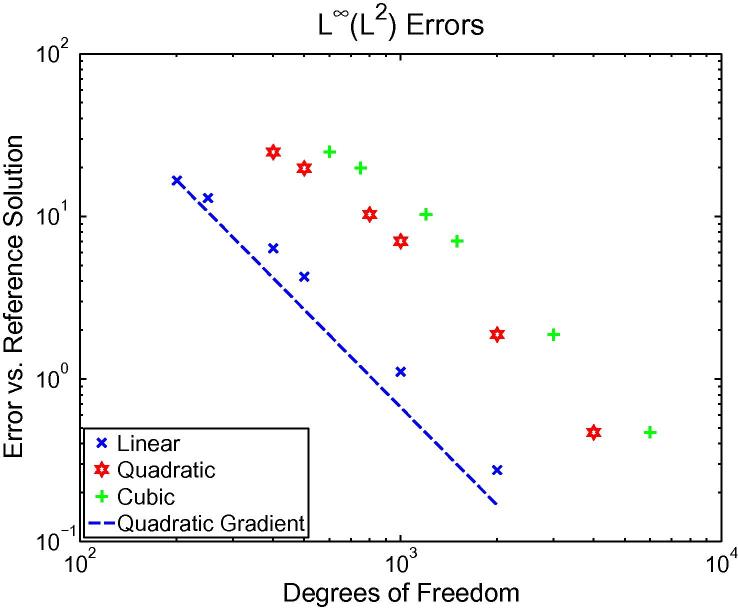
1D simulation results demonstrating that we never do better than quadratic convergence in the *L*^∞^(*L*^2^) norm when the approximation to *w* is linear-only, even when the finite element space *S*_*hp*_ is of high enough order to allow for better convergence. The points are the measured errors, the dashed line shows a theoretical quadratic convergence gradient. The simulation was 1D and the errors are measured against a reference with mesh spacing *h* = 0.0001 cm, whereas the test simulations use meshes with six different spacings ranging from *h* = 0.01 cm to *h* = 0.001 cm.

Taking $\overset{˜}{p}=1$ is not sufficient; the problem with this can be seen by considering the error in a 1D simulation at different levels of *h*-refinement with *p* kept fixed. Fig. 2 shows how the error in the *L*^∞^(*L*^2^) norm (maximum-in-time of the *L*^2^(*Ω*) spatial norm) varies with *h* using different finite element basis degrees *p* when $\overset{˜}{p}=1$, demonstrating the restricted convergence. Because of inequality (1) we might expect the error in this norm to be *O*(*h*^*p*+1^), but we only see *O*(*h*^2^). This is consistent with the quadratic convergence of the error in *w* caused by $\overset{˜}{p}=1$. Instead, we let $\overset{˜}{p}=p$ to allow for the full convergence rate. In the next subsection we put this on a solid theoretical foundation.

#### 2.3.1 Analysis for the error in *L*^2^

We begin with a necessary lemma, supposing that the true solution to system (2) has at least *k* derivatives.

Lemma 2.1 *For u* ∈ *H*^*k*^*(Ω), the L*^*2*^ *projection π* *:* *H*^*k*^*(Ω)* → *S*_*hp*_ *satisfies*

$$\|u-\pi u{\|}_{0\text{,}2}⩽{\mathrm{Ch}}^{\mu }p^{-k}\|u{\|}_{k\text{,}2}$$

*for k* > *3/2 and μ* *=* *min{p* *+* *1,k*}*.*

Proof A modification of Theorem 3 of [39] produces a continuous piecewise polynomial *ψ* on our mesh which satisfies

$$\|u-\psi {\|}_{0\text{,}2}⩽{\mathrm{Ch}}^{\mu }p^{-k}\|u{\|}_{k\text{,}2}$$

for quadrilateral meshes; the proof Theorem 4 of [40] contains the details necessary to modify *ψ* so that it applies when the mesh consists of triangles. Because *π* is the *L*^2^ projection, we have ∥*u* − *πu*∥_0,2_ ⩽ ∥*u* − *ψ*∥_0,2_. □

The importance of $\overset{˜}{p}$ can be seen via an *a priori* error estimate (see [41]). We examine this in what follows, where we work with the vectorised formulation of system (2), treating the spatial discretisation of the non-diffusing state variables in the finite element framework. To this end we introduce the inner product ${\left({\left[a_{1}a_{2}\right]}^{T}\text{,}{\left[b_{1}b_{2}\right]}^{T}\right)}_{F}≔(a_{1}\text{,}b_{1})+\sum_{i=1}^{m}(a_{2\text{,}i}\text{,}b_{2\text{,}i})$ and associated norm ∥ · ∥_*F*_, where (*a*, *b*) is the standard *L*^2^ inner product, *a*_1_, *b*_1_ ∈ *L*^2^(*Ω*) and *a*_2_, *b*_2_ ∈ (*L*^2^(*Ω*))^*m*^. Define

$$\Pi_{h\overset{˜}{p}}:H^{1}(\Omega )\times {\left(L^{2}(\Omega )\right)}^{m}\to {\left(S_{h\overset{˜}{p}}\right)}^{m+1}\text{,}$$

the *L*^2^(*Ω*) projection into $S_{h\overset{˜}{p}}$ in each of its *m* + 1 components, with *m* the number of components of *w*, and also the (*m* + 1) × (*m* + 1) matrix-like operator

$$G≔\left(\begin{array}{cccc} \nabla & 0 & \ldots & 0\\ 0 & 0 & \ddots & \vdots \\ \vdots & \ddots & \ddots & \vdots \\ 0 & \ldots & \ldots & 0 \end{array}\right)$$

which operates as *G***u** = *G*[*u w*]^*T*^ = [∇*u* 0]^*T*^.

Dropping the constants and conductivity tensor *σ* from system (2) for clarity, we obtain the weak form of the full system

(5) $${\left(u_{t}\text{,}\chi \right)}_{F}+{\left(Gu\text{,}G\chi \right)}_{F}={\left(f(u)\text{,}\chi \right)}_{F}\text{,}\;\forall \chi \in H^{1}(\Omega )\times {\left(L^{2}(\Omega )\right)}^{m}\text{,}$$

where **u**(*x*, *t*) ≔ [*u*(*x*, *t*) *w*(*x*, *t*)]^*T*^ and **f**(**u**) ≔ [*I*_*total*_(**u**) *g*(**u**)]^*T*^, with the symbols as in system (2). Let $p=(p\text{,}\overset{˜}{p})\text{,}\;u_{hp}(x\text{,}t)≔{\left[u_{\mathrm{hp}}(x\text{,}t)\;w_{h\overset{˜}{p}}(x\text{,}t)\right]}^{T}$ be the solution to our FEM formulation

(6) $${\left(u_{hp\text{,}t}\text{,}\chi \right)}_{F}+{\left(Gu_{h\text{,}p}\text{,}G\chi \right)}_{F}={\left(\Pi_{h\overset{˜}{p}}f(u_{hp})\text{,}\chi \right)}_{F}\text{,}\;\forall \chi \in S_{\mathrm{hp}}\times {\left(S_{h\overset{˜}{p}}\right)}^{m}\text{,}$$

where we are solving for *u* using elements of order *p* and for *w* using elements of order $\overset{˜}{p}$, and we have applied $\Pi_{h\overset{˜}{p}}$ to obtain an approximation in $S_{h\overset{˜}{p}}$ to the current *I*_*total*_ for the purpose of computationally-efficient matrix-based right-hand side assembly of the linear system (3).

Let $R_{hp}:H^{1}(\Omega )\times {\left(L^{2}(\Omega )\right)}^{m}\to S_{\mathrm{hp}}\times {\left(S_{h\overset{˜}{p}}\right)}^{m}$ be a Ritz-*L*^2^ projection, given by

(7a) ((G+I^)u,(G+I^)χ)F=((G+I^)Rhpu,(G+I^)χ)F∀χ∈Shp×(Shp˜)m,

(7b) $$\int_{\Omega }{\left(R_{hp}u\right)}_{1}{\mathrm{dx}}^{n}=0\text{,}$$

where I^ is the (*m* + 1) × (*m* + 1) identity matrix adjusted so that I^(1,1)=0,(z)1 denotes the first component of *z*, and Eq. (7b) ensures that *R*_*h***p**_ is well-defined. We note that in particular *R*_*h***p**_ satisfies

(8) $${\left(Gu\text{,}G\chi \right)}_{F}={\left({\mathrm{GR}}_{hp}u\text{,}G\chi \right)}_{F}\;\forall \chi \in S_{\mathrm{hp}}\times {\left(S_{h\overset{˜}{p}}\right)}^{m}\text{.}$$

Informed by [41], we now prove the following theorem on the error in the finite element approximation, which demonstrates the importance of $\overset{˜}{p}$.

Theorem 2.1 *Let* **u** *be the solution to system* (5) *with initial conditions as given in system* (2), *and* **u**_*h***p**_ *the solution to its semidiscrete-in-space form* (6)*. Let μ* *=* *min{p* *+* *1,* *k*} *and* $\overset{˜}{\mu }=\min\{\overset{˜}{p}+1\text{,}k\}$*, where* **u** *has spatial derivatives of order at least k. Suppose k* > *3/2 and that* **f** *is Lipschitz continuous in* **u** *with respect to the norm* ∥ · ∥_*F*_*. Then for some constant C, the following a priori estimate for the error at time T holds:*

(9) $$\|u_{hp}(T)-u(T){\|}_{F}⩽C\left(\|u_{hp}(0)-u(0){\|}_{F}+h^{\mu }p^{-k}\|u(0){\|}_{k\text{,}2}+h^{\overset{˜}{\mu }}{\overset{˜}{p}}^{-k}\overset{m}{\underset{i=1}{\sum }}\|w_{i}(0){\|}_{k\text{,}2}+\left[\int_{0}^{T}h^{\mu }p^{-k}\|u{\|}_{k\text{,}2}+h^{\overset{˜}{\mu }}{\overset{˜}{p}}^{-k}\overset{m}{\underset{i=1}{\sum }}\|w_{i}{\|}_{k\text{,}2}+\|(f-\Pi_{h\overset{˜}{p}}f)(u){\|}_{F}+h^{\mu }p^{-k}\|u_{t}{\|}_{k\text{,}2}+h^{\overset{˜}{\mu }}{\overset{˜}{p}}^{-k}\overset{m}{\underset{i=1}{\sum }}\|g_{i}{\|}_{k\text{,}2}\mathrm{dt}\right]+h^{\mu }p^{-k}\|u(T){\|}_{k\text{,}2}+h^{\overset{˜}{\mu }}{\overset{˜}{p}}^{-k}\overset{m}{\underset{i=1}{\sum }}\|w_{i}(T){\|}_{k\text{,}2}\right)\text{,}$$

Proof Following [41], we decompose the error we wish to bound as

$$u-u_{hp}=\underset{\rho }{\underset{︸}{u-R_{hp}u}}+\underset{\theta }{\underset{︸}{R_{hp}u-u_{hp}}}\text{,}$$

and then bound *ρ* and *θ* separately. *θ* satisfies

$${\left(\theta_{t}\text{,}\chi \right)}_{F}+{\left(G\theta \text{,}G\chi \right)}_{F}={\left({\left(R_{hp}u\right)}_{t}\text{,}\chi \right)}_{F}-{\left(u_{hp\text{,}t}\text{,}\chi \right)}_{F}+{\left({\mathrm{GR}}_{hp}u\text{,}G\chi \right)}_{F}-{\left(Gu_{hp}\text{,}G\chi \right)}_{F}\;\forall \chi \in S_{\mathrm{hp}}\times {\left(S_{h\overset{˜}{p}}\right)}^{m}\text{;}$$

applying Eq. (8) and rearranging,

$${\left(\theta_{t}\text{,}\chi \right)}_{F}+{\left(G\theta \text{,}G\chi \right)}_{F}=-{\left(u_{hp\text{,}t}\text{,}\chi \right)}_{F}-{\left(Gu_{hp}\text{,}G\chi \right)}_{F}+{\left(Gu\text{,}G\chi \right)}_{F}+{\left({\left(R_{hp}u\right)}_{t}\text{,}\chi \right)}_{F}\;\forall \chi \in S_{\mathrm{hp}}\times {\left(S_{h\overset{˜}{p}}\right)}^{m}\text{.}$$

The first two terms are replaced using the semidiscrete Eq. (6):

$${\left(\theta_{t}\text{,}\chi \right)}_{F}+{\left(G\theta \text{,}G\chi \right)}_{F}=-{\left(\Pi_{h\overset{˜}{p}}f(u_{hp})\text{,}\chi \right)}_{F}+{\left(Gu\text{,}G\chi \right)}_{F}+{\left({\left(R_{hp}u\right)}_{t}\text{,}\chi \right)}_{F}+{\left(f(u)\text{,}\chi \right)}_{F}-{\left(f(u)\text{,}\chi \right)}_{F}\;\forall \chi \in S_{\mathrm{hp}}\times {\left(S_{h\overset{˜}{p}}\right)}^{m}\text{.}$$

Using Eq. (5),

$${\left(\theta_{t}\text{,}\chi \right)}_{F}+{\left(G\theta \text{,}G\chi \right)}_{F}={\left(f(u)-\Pi_{h\overset{˜}{p}}f(u_{hp})\text{,}\chi \right)}_{F}-{\left(u_{t}\text{,}\chi \right)}_{F}+{\left({\left(R_{hp}u\right)}_{t}\text{,}\chi \right)}_{F}={\left(f(u)-\Pi_{h\overset{˜}{p}}f(u_{hp})\text{,}\chi \right)}_{F}+{\left({\left(R_{hp}u\right)}_{t}-u_{t}\text{,}\chi \right)}_{F}={\left(f(u)-\Pi_{h\overset{˜}{p}}f(u_{hp})\text{,}\chi \right)}_{F}-{\left(\rho_{t}\text{,}\chi \right)}_{F}\;\forall \chi \in S_{\mathrm{hp}}\times {\left(S_{h\overset{˜}{p}}\right)}^{m}\text{.}$$

Thus, we have

(10) $${\left(\theta_{t}\text{,}\chi \right)}_{F}+{\left(G\theta \text{,}G\chi \right)}_{F}={\left(f(u)-\Pi_{h\overset{˜}{p}}f(u_{hp})\text{,}\chi \right)}_{F}-{\left(\rho_{t}\text{,}\chi \right)}_{F}\;\forall \chi \in S_{\mathrm{hp}}\times {\left(S_{h\overset{˜}{p}}\right)}^{m}\text{.}$$

Taking *χ* = *θ* (which is possible because we chose *θ* such that $\theta \in S_{\mathrm{hp}}\times {\left(S_{h\overset{˜}{p}}\right)}^{m}$) and applying Cauchy–Schwarz,

$$\begin{array}{cc} & {\left(\theta_{t}\text{,}\theta \right)}_{F}+{\left(G\theta \text{,}G\theta \right)}_{F}={\left(f(u)-\Pi_{h\overset{˜}{p}}f(u_{hp})\text{,}\theta \right)}_{F}-{\left(\rho_{t}\text{,}\theta \right)}_{F}\text{,}\\ & \frac{1}{2}\frac{d}{\mathrm{dt}}\|\theta {\|}_{F}^{2}+\|\nabla \theta {\|}_{0\text{,}2}^{2}⩽(\|f(u)-\Pi_{h\overset{˜}{p}}f(u_{hp}){\|}_{F}+\|\rho_{t}{\|}_{F})\|\theta {\|}_{F}\text{,} \end{array}$$

or since $\|\nabla \theta {\|}_{0\text{,}2}^{2}⩾0$ and $\frac{d}{\mathrm{dt}}\|\theta {\|}_{F}^{2}=2\|\theta {\|}_{F}\frac{d}{\mathrm{dt}}\|\theta {\|}_{F}$,

$$\begin{array}{cc} & \frac{d}{\mathrm{dt}}\|\theta {\|}_{F}⩽\|f(u)-\Pi_{h\overset{˜}{p}}f(u_{hp}){\|}_{F}+\|\rho_{t}{\|}_{F}\text{,}\\ & \frac{d}{\mathrm{dt}}{\left\|\theta \right\|}_{F}⩽\|f(u)-\Pi_{h\overset{˜}{p}}f(u){\|}_{F}+\|\Pi_{h\overset{˜}{p}}f(u)-\Pi_{h\overset{˜}{p}}f(u_{hp}){\|}_{F}+\|\rho_{t}{\|}_{F}\text{.} \end{array}$$

Using the Lipschitz property of **f** and the *L*^2^ projection bound $\|\Pi_{h\overset{˜}{p}}(f(v)){\|}_{F}⩽\|f(v){\|}_{F}$,

$$\frac{d}{\mathrm{dt}}\|\theta {\|}_{F}⩽\|(f-\Pi_{h\overset{˜}{p}}f)(u){\|}_{F}+L\|u-u_{hp}{\|}_{F}+\|\rho_{t}{\|}_{F}\text{.}$$

Now we can integrate to obtain

$$\begin{array}{cc} & \|\theta (T){\|}_{F}⩽\|\theta (0){\|}_{F}+\int_{0}^{T}L\|u-u_{hp}{\|}_{F}+\|(f-\Pi_{h\overset{˜}{p}}f)(u){\|}_{F}+\|\rho_{t}{\|}_{F}\mathrm{dt}\text{,}\\ & \|\theta (T){\|}_{F}⩽\|\theta (0){\|}_{F}+\int_{0}^{T}L(\|\theta {\|}_{F}+\|\rho {\|}_{F})+\|(f-\Pi_{h\overset{˜}{p}}f)(u){\|}_{F}+\|\rho_{t}{\|}_{F}\mathrm{dt}\text{.} \end{array}$$

Applying Gronwall’s lemma, we see that

$$\left\|\theta (T){\|}_{F}⩽C(\|\theta (0){\|}_{F}+\int_{0}^{T}\|\rho {\|}_{F}+\|(f-\Pi_{h\overset{˜}{p}}f)(u){\|}_{F}+\|\rho_{t}{\|}_{F}\mathrm{dt}\right)$$

where *C* depends on *T* [41]. Using this estimate and the bounds

(11) $$\begin{array}{cc} & \|\rho (T){\|}_{F}⩽C\left(h^{\mu }p^{-k}\|u(T){\|}_{k\text{,}2}+h^{\overset{˜}{\mu }}{\overset{˜}{p}}^{-k}\overset{m}{\underset{i=1}{\sum }}\|w_{i}(T){\|}_{k\text{,}2}\right)\\ & \|\rho_{t}{\|}_{F}⩽C\left(h^{\mu }p^{-k}\|u_{t}{\|}_{k\text{,}2}+h^{\overset{˜}{\mu }}{\overset{˜}{p}}^{-k}\overset{m}{\underset{i=1}{\sum }}\|g_{i}{\|}_{k\text{,}2}\right) \end{array}$$

from [15] and Lemma 2.1, where *k* can be as large as is allowed by the smoothness of *u* and *w*. By combining the bounds for *θ* and *ρ* together with

$$\|\theta (0){\|}_{F}⩽\|u_{hp}(0)-u(0){\|}_{F}+\|R_{hp}u(0)-u(0){\|}_{F}⩽\|u_{hp}(0)-u(0){\|}_{F}+{\mathrm{Ch}}^{\mu }p^{-k}\|u(0){\|}_{k\text{,}2}+{\mathrm{Ch}}^{\overset{˜}{\mu }}{\overset{˜}{p}}^{-k}\overset{m}{\underset{i=1}{\sum }}\|w_{i}(0){\|}_{k\text{,}2}\text{,}$$

which can essentially be found in [41], we arrive at the estimate for the full error:

$$\|u_{hp}(T)-u(T){\|}_{F}\;⩽\;C\left(\|u_{hp}(0)-u(0){\|}_{F}+h^{\mu }p^{-k}\|u(0){\|}_{k\text{,}2}+h^{\overset{˜}{\mu }}{\overset{˜}{p}}^{-k}\overset{m}{\underset{i=1}{\sum }}\|w_{i}(0){\|}_{k\text{,}2}+\left[\int_{0}^{T}h^{\mu }p^{-k}\|u{\|}_{k\text{,}2}+h^{\overset{˜}{\mu }}{\overset{˜}{p}}^{-k}\overset{m}{\underset{i=1}{\sum }}\|w_{i}{\|}_{k\text{,}2}+\|(f-\Pi_{h\overset{˜}{p}}f)(u){\|}_{F}+h^{\mu }p^{-k}\|u_{t}{\|}_{k\text{,}2}+h^{\overset{˜}{\mu }}{\overset{˜}{p}}^{-k}\overset{m}{\underset{i=1}{\sum }}\|g_{i}{\|}_{k\text{,}2}\mathrm{dt}\right]+h^{\mu }p^{-k}{\left\|u(T)\right\|}_{k\text{,}2}+h^{\overset{˜}{\mu }}{\overset{˜}{p}}^{-k}\overset{m}{\underset{i=1}{\sum }}\|w_{i}(T){\|}_{k\text{,}2}\right)\text{,}$$

as required. □

The theorem demonstrates that the approximation properties of $S_{h\overset{˜}{p}}$ are crucial. For example, with *p* = 3 we would like to get a quartic convergence rate of *u*_*hp*_ to *u* at any particular time *T* in the norm ∥*u*_*hp*_ − *u*∥_0,2_ as we refine *h*, but we can not expect this if $\overset{˜}{p}=1$ because of the *O*(*h*^2^) terms that then appear in inequality (9). This agrees with our experimental results (see Fig. 2).

### 2.4 Proper treatment of the cell model PDEs for high order FEM

For an element *τ*, a nodal basis ${\left\{\phi_{i}^{L}\right\}}_{i=1}^{N(p)}$ of degree *p* (for the space of polynomials of degree  ⩽ *p* on *τ*) associated with a set of nodes ${\left\{x_{i}\right\}}_{i=1}^{N(p)}\text{,}x_{i}\in \tau \text{,}$ is such that $\phi_{i}^{L}(x_{j})=\delta_{\mathrm{ij}}\;\forall i\text{,}j$ [36]. For our problem (2), we use a nodal basis of degree $\overset{˜}{p}$ on the elements of M to approximate *w*; the Gauss–Lobatto points associated with polynomials of order $\overset{˜}{p}$ [42] make a good choice here for high-quality approximation. This naturally gives us an approximation *ι* to $\Pi_{h\overset{˜}{p}}$; we compute the current at each *x*_*i*_, and we immediately have the nodal basis weights for our degree-$\overset{˜}{p}$ projection *ιI*_*total*_, interpolating *I*_*total*_ at the points *x*_*i*_. Informed by Theorem 2.1, we take $\overset{˜}{p}⩾p$ to obtain the expected convergence rate for our choice of *p*.

If we assume that the *L*^2^ projection $\Pi_{h\overset{˜}{p}}$ used in Theorem 2.1 and the interpolation operator *ι* that we replace it by in practice are sufficiently close, we can suppose that the error between **f** and *ι***f** is $O(h^{\overset{˜}{\mu }}p^{-k})$, where we also assume sufficient smoothness of **f**. Of course, we cannot be certain that this error will be achieved with our scheme given that we have not attempted to carefully approximate $\Pi_{h\overset{˜}{p}}$, but our experimental work has indicated that *ι* is sufficient.

In order to use the product ***M***(*I*_1_, … , *I*_*N*_) to efficiently integrate the current, because ***M*** uses a hierarchical basis, on each iteration of our simulation we change the basis *ιI*_*total*_ is represented in from nodal to hierarchical.

Because there are no spatial derivatives in the PDE for *w*, the nodal finite element system reduces to effectively a large number of local ODE systems; these are familiar in concept as the pointwise ODE models that occur in standard linear FEM approaches to this problem. In practice, this means that we never have to form matrices for the nodal system.

### 2.5 Discontinuities in the cell model

This subsection describes a deficiency in the cell model which must be overcome for a proper high-order FEM implementation. Many of the cardiac cell models in the literature include some discontinuous functions which describe the voltage-dependent rate at which conductive ion channels embedded in the cell membrane open and close [43]. Such issues can prevent high-order numerical schemes from attaining their theoretical rates of convergence. This has been noted previously for high order temporal schemes [43]; here we identify it as a problem for spatial schemes also. Fig. 4(a) shows the problem; note the deviation of the quartic and cubic solutions from their respective theoretical convergence gradients. The discontinuity exists because two different analytic expressions have been fitted to experimental data for the voltage-dependent transition rates for the ion channel gates in the cell model. Which of these expressions is used is determined by the transmembrane potential, with the discontinuity at the point where the model switches between them (−40 mV).

A continuous replacement has been around for some time [34] but has not been adopted; the problem has propagated due to the fact that some cell models have been created as modifications older ones. Introducing this continuous form is required to ensure theoretically optimal errors in the solutions to system (2). Compare Fig. 4(a) with Fig. 4(b) which differs only in that the cell model used in Fig. 4(b) has undergone this modification.

**Fig. 3:**
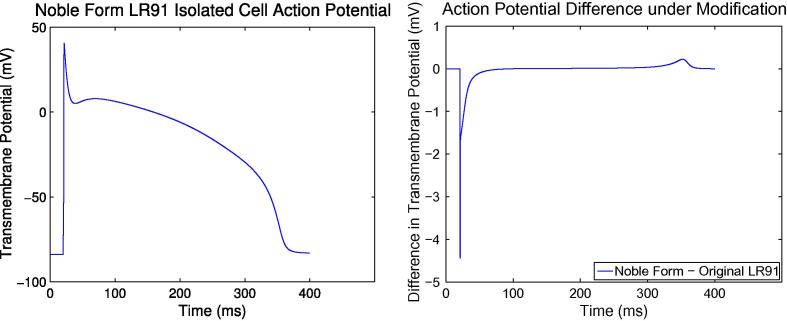
Plot comparing the action potential in an isolated cell model using the standard Luo-Rudy 1991 formulation and the Noble-form modification. Left: Noble-form modified LR91 action potential in an isolated cell. On this scale, differences caused by the modification would be hardly noticeable. Right: original LR91 transmembrane potential subtracted from Noble-form LR91 transmembrane potential. Note that the scales on the y-axes differ.

Fig. 3 shows the AP difference between the standard LR91 model and the modified Noble-form LR91; they are quite minor. Given the fact that cell models are generated from experimental data naturally prone to experimental error [44], these differences are probably not worth being concerned with, especially given that the discontinuities do not appear to be biologically justified. We must check for and remove such discontinuities when using a particular cell model for simulation.

### 2.6 Including a smooth fibre field

In one of our test simulations (see Section 3.3.2), we use a geometry that includes holes representing blood vessels passing through the tissue (see Fig. 7(b)). In order to construct a realistic conductivity tensor *σ* we generate fibre orientation vector fields using a Laplace–Dirichlet approach [45]. This involves solving Laplace’s equation on the domain with Dirichlet boundary condition +1 on one external edge of *Ω*, −1 on the opposite external edge and zero Neumann conditions on all other boundaries. The result is a conductivity tensor field which approximates the way that cardiac fibres negotiate around blood vessels [46].

## 3 Simulations

All simulations were performed in MATLAB and use a semi-implicit backward Euler time discretisation scheme. We use a fixed $\overset{˜}{p}=4$ regardless of the value of *p*(⩽4) so that we can focus on the effect of varying the approximation order for the transmembrane potential *u*; leaving $\overset{˜}{p}$ fixed means that we can examine this in a fair manner. Timings presented are for a 3.4 GHz CPU.

### 3.1 Convergence in 1D

**Fig. 4:**
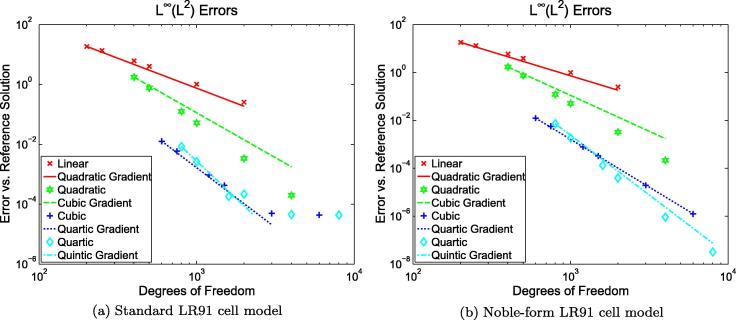
Data from 20 ms simulations on a 2 cm 1D domain using our method with and without the Noble-form LR91 cell model modification. Note the limited accuracy caused by the discontinuity in standard LR91. Δ*t* = 0.001 ms. *L*^∞^(*L*^2^) is the maximum-in-time of the *L*^2^-norm of the error in space. The errors are against a quartic reference solution with *h* = 10^−4^ cm.

**Fig. 5:**
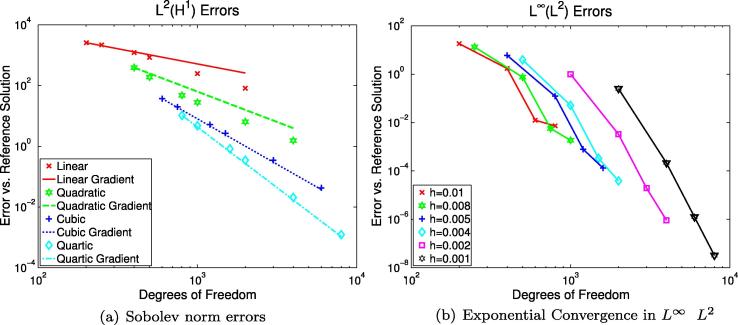
Noble-form LR91 with our method. Fig. 5(a) shows the *L*^2^(*H*^1^) norm of the error; this is the *L*^2^-in-time norm of the Sobolev ∥ · ∥_1,2_ norm of the error in space. Fig. 5(b) shows the same data as Fig. 4(b), but with the exponential convergence in *p* highlighted instead. Data from 20 ms simulations on a 2 cm 1D domain. Δ*t* = 0.001 ms. The errors are against a quartic reference solution with *h* = 10^−4^ cm. The values of *h* given are in cm.

We stimulated a 2 cm 1D domain at one end with a ramp stimulus using a time-step of Δ*t* = 0.001 ms and simulated the first 20 ms of activation. The errors in two different norms are presented in Fig. 4(b) and Fig. 5(a) and use as a reference a quartic solution generated with *h* = 0.0001 cm. Fig. 5(a) shows the error measured using the *L*^2^-in-time norm of the $H^{1}(\Omega )$ norm in space, for which we expect *O*(*h*^*p*^) convergence gradients [15]. Note the agreement of Figs. 4(b) and 5(a) with the theoretical error gradients presented, and the limited accuracy displayed in Fig. 4(a) caused by the discontinuities in the standard LR91 cell model. The exponential error convergence rate achievable using *p*-refinement is emphasised in Fig. 5(b). Note that we use a conductivity of 0.5 S m^−1^ for all our convergence figures.

In order to perform a robust investigation of conduction velocity (CV), we used a 6 cm domain, stimulated at one end with a ramp stimulus and used Δ*t* = 0.01 ms; the activation time for the node at the opposite end of the domain and the CVs are presented in Table 1. We performed two sets of simulations in order to gather data on the fast and slow conductivities that we use later, respectively parallel and perpendicular to the fibres in anisotropic 2D simulations. Note how small *h* needs to be when using linear elements to achieve CV convergence.

### 3.2 2D homogeneous conductivity

Having demonstrated the superior accuracy in 1D, we move on to 2D. In this subsection, unless otherwise noted we use a time-step Δ *t* = 0.01 ms.

**Fig. 6:**
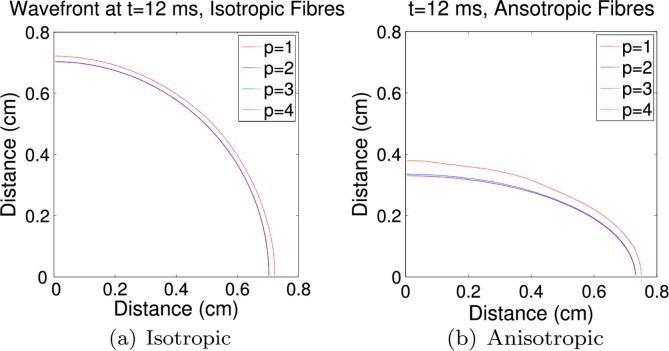
Wavefront location at *t* = 12 ms with *p* = 1–4, demonstrating the scheme with isotropic tissue and with fibres aligned with the *x*-axis having anisotropic conductivity (1 S m^−1^ along the fibres and 0.2 S m^−1^ perpendicular to them). In both cases, the larger *p* is, the less distance the wavefront has propagated. Note that *p* = 2–4 are indistinguishable in (a), and that *p* = 3 and *p* = 4 are indistinguishable in (b). This simulation used Δ*t* = 0.01 ms, the mean element diameter was 0.0113 cm and the stimulus was applied to the lower-left corner of the domain.

Before performing our experiments, we demonstrate the effect of anisotropy in our simulations. Fig. 6 shows wavefront locations (*u* = 0 mV) with *p* = 1–4 for two simulation modes at 12 ms, one with homogeneous isotropic conductivity (Fig. 6(a)) and one with homogeneous anisotropic conductivity using fibres aligned to the *x*-axis (Fig. 6(b)). In the latter case, the conductivity along the fibres is the same as that for the isotropic case (1 S cm^−1^), and perpendicular to the fibres we use one-fifth of that value. Both simulations were initiated with the same stimulus current in the lower-left corner of the domain and use a mesh with mean element diameter 0.0113 cm. Note the poor accuracy of the linear case, and note further that where the anisotropic *p* = 1 wavefront has propagated predominantly perpendicular to the fibres in Fig. 6(b), its accuracy is even worse. This is due to the low conduction velocity in that direction, requiring better approximation properties (smaller elements, larger *p*) to properly capture *u* at the wavefront.

In our first 2D experiment we investigated a 1 cm by 1 cm 2D domain with homogeneous isotropic conductivity. A ramp stimulus was applied to a rectangular region along the bottom of the domain, generating a planar propagating wave. The measured CVs and activation times at the top-right corner of the domain are given in Table 2. Each simulation was performed with Δ*t* = 0.01 ms and Δ *t* = 0.001 ms (asterisked) in order to investigate how the temporal error affects the results. The CV is an average taken over many randomly selected point pairs in the domain. Percentage errors in conduction velocity are also presented in the table, using the finest quartic simulation with the same value of Δ*t* as a reference. Note the high accuracy of the quartic simulations, and the minimal variation in the percentage errors caused by varying Δ*t*. Details on the degrees of freedom are in Table 5.

The data shows that *p* = 4 on the coarsest mesh or *p* = 3 with Δ*t* = 0.01 ms on the second coarsest mesh ought to be preferred over *p* = 1 on the finest mesh, given that this produces at worst a halving of the percentage error using a linear system which takes less than half the time to solve. Alternatively, *p* = 2 on the second coarsest mesh with Δ*t* = 0.01 ms produces roughly equivalent accuracy to the finest linear solution, but does so using a linear system which can be solved eight times faster.

### 3.3 2D inhomogeneous conductivity

#### 3.3.1 Plain square domain with cubic fibre field

**Fig. 7:**
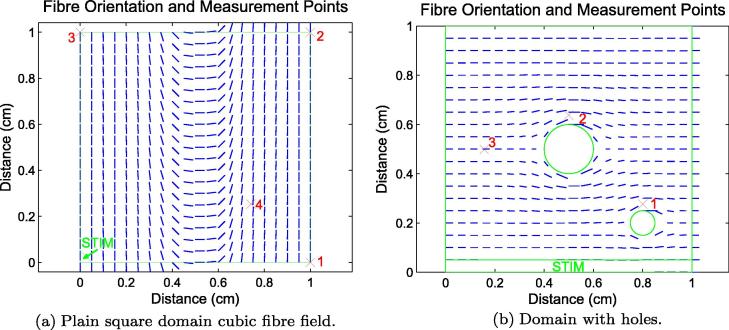
Fibre orientations used for the inhomogeneous simulations. The marked points are those at which activation time is measured. For Fig. 7(a), results are displayed in Table 3 and stimulation was a ramp in the bottom-left corner. For Fig. 7(b), results are displayed in in Table 4 and stimulation was a ramp along the bottom of the domain.

We studied simulation on a 1 cm by 1 cm domain using Δ*t* = 0.01 ms and inhomogeneous anisotropic conductivity, with the conductivity along the fibres the same as that used in the isotropic case above (1 S m^−1^), and one-fifth of this value in the perpendicular direction. The fibre field is defined by a cubic polynomial designed to represent cardiac fibres rapidly changing orientation (see Fig. 7(a)) and is integrated by reading the value of the vector field at each Gauss point [47] during matrix construction. The domain was stimulated with a ramp in the bottom-left corner of the domain. The measured activation times at the four points shown in Fig. 7(a) are given in Table 3. Note how cubic and quartic elements display superior accuracy to all tested linear simulations by the second coarsest level. Details on the degrees of freedom are in Table 5.

#### 3.3.2 Domain with holes

**Fig. 8:**
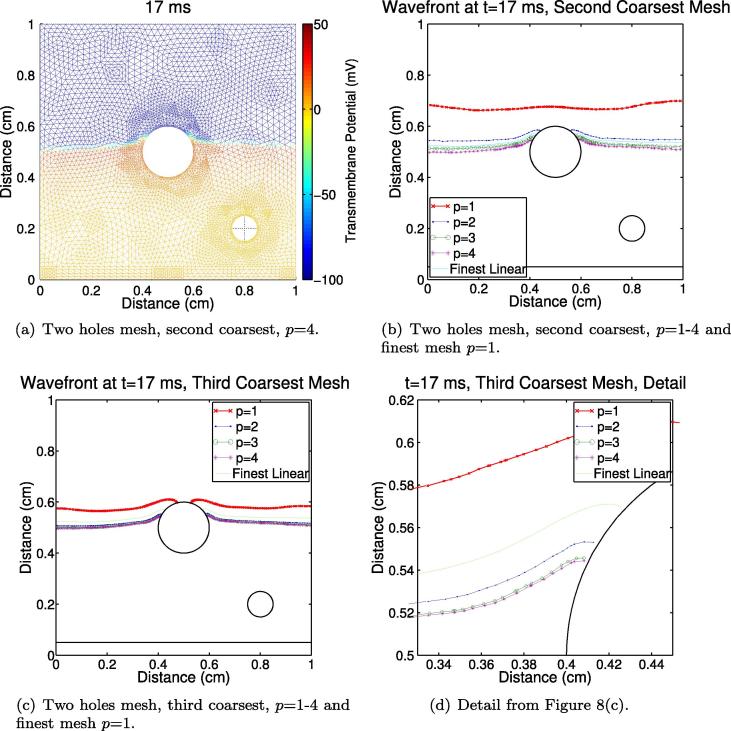
Wavefront location at 17 ms using various meshes and degrees. (a) Transmembrane potential distribution on the second coarsest mesh with *p* = 4. Note that each triangle represents a single element of the mesh, and that this is independent of *p*. (b) Wavefront (*u* = 0) contours on the second coarsest mesh with various degrees compared to *p* = 1 on the finest mesh. (c) Wavefront contours on the second coarsest mesh with various degrees compared to *p* = 1 on the finest mesh. (d) Detail from Fig. 8(c). See the corresponding activation time data in Table 4.

Fig. 7(b) shows the fibre vector field on a 1 cm by 1 cm domain with two holes representing blood vessels passing through the simulation plane. We performed this study to demonstrate the applicability of the method when the domain contains fine structure that must be properly captured using small elements. This vector field was generated using the Laplace–Dirichlet approach and the time-step used was Δ*t* = 0.01 ms. The activation times at the three points shown in Fig. 7(b) are given in Table 4. Our results show that the second coarsest mesh with *p* = 3 can half the worst percentage error in activation time using a linear system which can be solved twice as quickly when compared to *p* = 1 on the finest mesh, or with *p* = 4, we can reduce the error to one-tenth that of *p* = 1 on the finest mesh with a linear system which takes slightly longer to solve. See Fig. 8 for some wavefront locations using various meshes and values of *p*; note how poor the wavefront location can be when *p* = 1 even on highly refined meshes, and how on the second coarsest mesh with *p* = 3 the results are better than the computationally twice-as-demanding *p* = 1 on the finest mesh.

### 3.4 Degrees of freedom

Details on the degrees of freedom for the various meshes and basis degrees are presented in Table 5.

## 4 Discussion and conclusion

### 4.1 Summary of results

We have shown how to successfully employ high-order finite element methods for simulation of the cardiac monodomain system (2), meeting or exceeding theoretical error convergence rates. The method achieves our goal of improved efficiency over linear finite elements; we produce highly accurate numerical solutions cheaply, as can be seen for the homogeneous conductivity by comparing the finest linear solution to the second coarsest cubic Δ*t* = 0.01 ms solution in Table 2. In this case, taking the finest quartic solution as a reference, we see that we obtain a six times smaller percentage error in activation time with a linear system that takes one-third of the time to solve. It can be used with isotropic and anisotropic conductivity, and geometries which include microstructure such as blood vessels passing through the tissue. The improved efficiency is key because good numerical approximations can take days to obtain with present technology.

As shown in Table 2, convergence in the conduction velocity requires very fine meshes when working with *p* = 1. Even at *h* = 0.0111 cm, the CV error is still around 2.5%; at that level, the error in the position of the wavefront will become very large during long-time, whole-heart (large domain) simulations.

In the case of inhomogeneous conductivity (Table 3) the second coarsest cubic solution is the point at which the accuracy starts to beat that of the finest linear solution. We note that in this case, the coarsest mesh should not be used for simulation; due to the low conductivity perpendicular to the fibre direction (one-fifth of that parallel to the fibres), we see that the activation times at nodes one and two overshoot the converged activation times. This is due to the effective mesh size being coarser when the conductivity is lower, introducing error into the solution. This explanation is supported by the fact that node three, being connected to the stimulus site by a straight line to which all the fibres along its length are aligned, does not experience any activation time problems. See also Table 1, which shows that the same effect occurs in 1D on very coarse meshes when using a low conductivity equal to that for the slow direction here (see *h* = 0.1, *p* = 4). Because error due to the coarse mesh discretisation of *u* will be compensated for by the accuracy gained as we increase *p*, we believe that this effect is due to insufficient resolution in the spatial discretisation of *w*, as this does not change as we refine *p*. Further investigation is needed to confirm this.

When holes are present in the domain, the simulations are much slower (Table 4) due to the increased number of elements required to mesh around the holes (see Fig. 8). Here we recommend using, for example, *p* = 2 on the third coarsest mesh for a sixfold accuracy improvement using a linear system which can be solved in half the time when compared to the finest mesh with *p* = 1. This accuracy can be seen by the position of the wave fronts in Figs. 8(c) and 8(d). Alternatively, the second coarsest *p* = 2 simulation can be seen as providing considerable accuracy improvement over *p* = 1 on the third coarsest mesh with a linear system which takes the same amount of time to solve. An example high-resolution anatomically-derived heart mesh has *h* = 0.0125 cm [33]; this is comparable with the third coarsest mesh here. See Table 6 for more detail and other possible choices of mesh and *p*.

### 4.2 Limitations on error reduction

We note that Figs. 4(b) and 5(b) show some unexpected behaviour, apparent in Fig. 4(b) as a crossing over of the errors for the *p* = 3 and *p* = 4 solutions, and in Fig. 5(b) as a smaller-than-expected error reduction when going from *p* = 3 to *p* = 4 on the coarser meshes.

We are not certain of the cause of this. Possible explanations include it being caused by the error introduced by our approximation of $\Pi_{h\overset{˜}{p}}$ by *ι*, or that the smoothness *k* of the cell model is limiting, for example where Lemma 2.1 is applied. Simulations using the very simple FitzHugh–Nagumo model [48] instead of LR91 with $\overset{˜}{p}=4$ do not display any problems, neither do $\overset{˜}{p}=6$ simulations with LR91 for *p* = 1–4.

Regardless of this, the efficiency remains compelling, and we note that the time-integrated Sobolev norm spatial error (Fig. 5(a)) does not appear to be so strongly affected.

### 4.3 Assumptions on the regularity of the cell model

Note that the requirement in Theorem 2.1 that **f** be Lipschitz in **u** is not satisfied for general cell models. However, if we suppose that **u** is bounded, the Lipschitz property will hold. For simulations, there is evidence to suggest this boundedness occurs for cell models modified to take electroporation currents into account [49–51]. Indeed, if we do not have boundedness, we can say that there is a problem in the simulation study design rather than in the numerics; it is certain that real-world quantities such as the transmembrane potential will be bounded in general, and all the more stringently whilst the cell is still functional. We also need to assume that the [Ca^2+^] is bounded away from zero; again this is likely to be the case in a functioning cell due to leak currents.

### 4.4 Representation of *I*_*total*_

The approach that we take to *I*_*total*_ could be compared to a more standard scheme which used a piecewise linear *I*_*total*_. Neither approach involves making any statements about what *I*_*total*_ looks like; even though *I*_*total*_ is smooth on elements and has discontinuous derivative at element boundaries, the positioning of the elements themselves is essentially arbitrary within the domain. It is clear therefore that no claims are really being made about any lack of smoothness in *I*_*total*_. Therefore, the assertion that we make in using the *p*-version that the smoothness of *u* (depending on the smoothness of *I*_*total*_) is sufficient so as not to limit the rate of convergence the *p*-version is not really any different from the assumptions that are implicitly made when discretising *Ω* for any standard application of FEM to the cardiac equations.

### 4.5 Gauss points for the cell model

Another approach to approximating the cell model variables *w* would be to construct a nodal basis using the Gauss points [47]. Instead of integrating the right-hand side of Eq. (3) using the mass matrix, we could do soby computing the current at each node and then integrating it against each basis function on each element using quadrature. This would work, but would be considerably more computationally demanding than the matrix-based assembly we use [52].

Another alternative would be to use the same nodal basis for *u* and for *w*; this would allow matrix-based assembly, but without the need for the change of basis (see Section 2.4). However, without the use of the hierarchical basis for *u*, our method would no longer be suitable for the *p*-adaptivity that we wish to investigate in future work.

### 4.6 Parallel simulation and timings

We note that while we have presented timings for both solving the linear system and also for all code that runs on each iteration (the difference between the two being primarily due to the cell model updates), less attention should be paid to the all-code timings. This is because our simulations were performed on one processor, and the cell state variable PDEs can be parallelised straightforwardly. As the future of cardiac simulation will be on massively parallel machines, this is important.

A consideration for implementing the approach presented in this work in a parallel computing environment is that as much of the work as possible should be achievable using data local to each element or patch of elements so that when the workload is divided up between different processors, the communication between them can be kept to a minimum. Our approach satisfies this requirement; for example, the change of basis that we perform to switch the current representation from nodal to hierarchical is performed locally on each element. In fact, the cell model and change of basis code could in future be implemented as GPU code; this has promise as some workers have already demonstrated that considerable speed-up is possible [53,54].

Timings in general should be treated with some caution, as there are many factors which can affect them. At the most basic level, solving the linear system using an iterative method requires matrix–vector multiplications (matvecs). With the sparse data structures used in this sort of problem, the cost of each matvec is a function of the number of nonzero entries in each row of the system matrix and of the number of rows it has; note that the last two vary with *h* and *p*. Each solve will require multiple matvecs to achieve convergence, with the number needed varying with the condition number of the system matrix. Thus, in addition to depending on *h* and *p*, the relative time costs of solving different linear systems will vary according to the preconditioner, the hardware type (parallel architecture, CPU cache size) and the software implementation. In this work, we have not looked at all of these; for example, further study investigating efficient preconditioning [29,7,8] for the linear system we have constructed may lead to further overall efficiency gains.

### 4.7 Conclusion

Because of the efficiency we have demonstrated, our work leads us to recommend preferring higher-order finite elements for cardiac monodomain simulation. With careful choice of meshes, this has the potential to reduce the amount of time required to perform whole-heart simulations; these have linear systems which are many times larger than the ones for our relatively small test simulations, so the overall efficiency savings will likely be substantial. Across all meshes, the advantages of using high *p* are considerable. In the case of anisotropic conductivity, it is likely that obtaining even larger gains will be possible by using our method together with meshes which are finer perpendicular to the fibre orientation than parallel to it in order to compensate for the reduced conduction velocity in that direction.

A recent paper comparing the convergence properties of eleven monodomain solvers [55] introduced a benchmark problem which can be easily applied to new codes so that they can be robustly evaluated relative to existing software. In future work, we will further evaluate our method by applying it to this problem.

Our one-and two-dimensional work using the monodomain should be seen as a proof-of-concept; in the future we shall look to extend this to three dimensions, bidomain and realistic cardiac geometry. The hierarchical nature of the elements we use lends them to adaptive methods which have the potential to bring about further significant gains in simulation efficiency.

## Figures and Tables

**Table 1:** Six cm 1D domain simulation with a ramp stimulus at one end and Δ*t* = 0.01 ms. The activation time is the time until the transmembrane potential at the node at the opposite end of the domain (at 6 cm) passes up through 0 mV. CVs are computed using the nodes at 2 and 4 cm.

Element diameter *h* (cm)	Basis degree *p*	Conductivity = 1 S m^−1^		Conductivity = 0.2 S m^−1^	
Activation time (ms)	CV (cm s^−1^)	Activation time (ms)	CV (cm s^−1^)
0.1	1	50.43	118.13	62.76	94.65
2	67.01	88.50	103.18	57.18
3	80.94	73.13	182.44	32.09
4	88.62	66.73	243.44	24.11

0.05	1	68.33	86.81	104.77	56.34
2	81.35	72.81	143.15	41.17
3	88.68	66.73	177.89	33.10
4	90.39	65.47	198.47	29.65

0.02	1	83.92	70.55	157.40	37.44
2	90.61	65.30	185.18	31.81
3	91.65	64.54	199.50	29.52
4	91.66	64.54	202.17	29.12

0.01	1	89.36	66.20	183.75	32.06
2	91.59	64.58	200.79	29.33
3	91.67	64.52	204.10	28.84
4	91.67	64.52	204.16	28.84

0.005	1	91.08	64.96	197.83	29.77
2	91.67	64.54	203.93	28.87
3	91.67	64.52	204.22	28.83
4	91.67	64.52	204.22	28.83

0.002	1	91.58	64.60	203.15	28.98
2	91.67	64.52	204.21	28.83
3	91.67	64.52	204.22	28.83
4	91.67	64.52	204.22	28.83

0.001	1	91.65	64.54	203.95	28.86
2	91.67	64.52	204.22	28.83
3	91.67	64.52	204.22	28.83
4	91.67	64.52	204.22	28.83

**Table 2:** Activation times, conduction velocities, percentage conduction velocity errors and linear system solve times for a variety of 2D meshes of *Ω* = [0, 1] × [0, 1] cm. The first timings presented are for a single time-step only and use preconditioned conjugate gradients (PCG) with an incomplete LU (ILU) decomposition of the portion of the system matrix corresponding to the linear basis functions as a preconditioner, and the second times all code on a time-step iteration (ILU PCG + cell updates, etc.). The stimulus was along the bottom edge of the domain and the activation time is for the node in the top-right corner. Asterisked basis degrees indicate that the simulation was run with Δ *t* = 0.001 ms instead of the usual Δ*t* = 0.01 ms. The percentage errors use as a reference the *p* = 4 simulation on the finest mesh with the same value of Δ*t*.

Mean element diameter *h* (cm)	Basis degree *p*	Activation time (ms)	CV (cm s^−1^)	% CV error vs. best solution	ILU PCG time (s)	Mean it. time (s)
0.0444	1	12.46	81.95	26.39	0.005	0.053
2	14.68	69.05	6.49	0.015	0.063
3	15.29	66.26	2.19	0.036	0.082
4	15.60	65.00	0.25	0.126	0.174
1^∗^	12.30	83.14	27.04	0.005	0.054
2^∗^	14.50	69.88	6.78	0.012	0.061
3^∗^	15.12	66.96	2.32	0.027	0.075
4^∗^	15.45	65.58	0.22	0.076	0.124

0.0222	1	14.36	70.56	8.82	0.016	0.201
2	15.51	65.41	0.88	0.045	0.232
3	15.64	64.91	0.11	0.112	0.299
4	15.65	64.84	0.00	0.304	0.493
1^∗^	14.18	71.41	9.13	0.016	0.209
2^∗^	15.34	66.06	0.95	0.042	0.229
3^∗^	15.48	65.51	0.11	0.095	0.285
4^∗^	15.50	65.44	0.00	0.202	0.393

0.0111	1	15.27	66.41	2.42	0.047	0.886
2	15.64	64.88	0.06	0.170	1.008
3	15.65	64.84	0.00	0.386	1.216
4	15.65	64.84	0.00	1.074	1.905
1^∗^	15.10	67.08	2.51	0.052	0.902
2^∗^	15.49	65.50	0.09	0.157	1.007
3^∗^	15.50	65.44	0.00	0.336	1.225
4^∗^	15.50	65.44	0.00	0.696	1.562

0.0055	1	15.55	65.22	0.59	0.368	3.866
2	15.65	64.84	0.00	0.909	4.513
3	15.65	64.84	0.00	2.103	5.643
4	15.65	64.84	0 (by def.)	6.066	9.621
1^∗^	15.40	65.86	0.64	0.195	3.811
2^∗^	15.50	65.45	0.02	0.573	4.331
3^∗^	15.50	65.44	0.00	1.225	4.896
4^∗^	15.50	65.44	0 (by def.)	2.782	6.455

**Table 3:** Activation times in the 1 cm by 1 cm domain with fibre orientation and four measurement points shown in Fig. 7(a) using various *h* and *p* values.

Mean element diameter *h* (cm)	Basis degree *p*	Activation time 1 (ms)	Activation time 2 (ms)	Activation time 3 (ms)	Activation time 4 (ms)
0.0452	1	18.29	22.64	13.17	15.68
2	24.55	29.03	15.12	20.51
3	27.89	31.86	15.66	22.88
4	35.86	37.63	16.04	28.65

0.0226	1	23.60	28.07	14.90	19.77
2	28.52	32.74	15.93	23.42
3	30.01	33.95	16.06	24.48
4	30.72	34.46	16.09	24.99

0.0113	1	27.52	31.77	15.72	22.71
2	30.38	34.22	16.08	24.74
3	30.75	34.47	16.09	24.98
4	30.80	34.50	16.09	25.02

0.0057	1	29.68	33.61	15.99	24.25
2	30.77	34.48	16.09	24.99
3	30.81	34.50	16.09	25.02
4	30.81	34.50	16.09	25.02

**Table 4:** Activation times in the 1 cm × 1 cm domain with holes, fibre orientation and three measurement points shown in Fig. 7(b) using various *h* and *p* values. The meshes are identified by maximum element diameter because the mean element size would convey little information due to the very small elements near the holes. The timings presented are for the linear system only (ILU PCG) and for the whole-timestep (ILU PCG + cell updates, etc.). More information about the meshes used is presented in Table 5, and some plots of the wavefront at 17 ms are shown in Fig. 8.

Max. element diameter *h* (cm)	Basis degree *p*	Activation time 1 (ms)	Activation time 2 (ms)	Activation time 3 (ms)	ILU PCG time (s)	Mean it. time (s)
0.0593	1	6.46	13.48	10.01	0.010	0.116
2	7.62	17.17	13.69	0.032	0.136
3	7.96	18.68	15.59	0.088	0.193
4	8.15	20.66	18.64	0.297	0.402

0.0296	1	7.24	16.14	13.08	0.022	0.319
2	8.00	18.84	15.96	0.068	0.350
3	8.12	19.54	16.86	0.176	0.456
4	8.14	19.85	17.29	0.571	0.855

0.0148	1	7.71	18.11	15.36	0.072	1.081
2	8.11	19.68	17.08	0.203	1.227
3	8.14	19.86	17.31	0.514	1.522
4	8.15	19.89	17.35	1.895	2.992

0.0076	1	7.96	19.21	16.64	0.410	4.532
2	8.14	19.86	17.32	1.049	4.876
3	8.15	19.89	17.35	2.415	6.427
4	8.15	19.89	17.35	9.430	13.391

**Table 5:** Table showing the degrees of freedom used throughout this study.

Mesh refinement level	Basis degree *p*	Degrees of freedom with		
Homogeneous conductivity	Inhomogeneous conductivity	Two holes
0	1	786	733	1770
2	3047	2841	6764
3	6784	6325	14,981
4	11,997	11,185	26,421

1	1	3,047	2,841	4,500
2	11,997	11,185	17,596
3	26,851	25,033	39,287
4	47,609	44,385	69,573

2	1	11,997	11,185	14,482
2	47,609	44,385	57,352
3	106,837	99,601	128,609
4	189,681	176,833	228,253

3	1	47,609	44,385	52,199
2	189,681	176,833	207,854
3	426,217	397,345	466,964
4	757,217	705,921	829,529

**Table 6:** Simulations on the mesh with holes in the context of the prescribed error tolerances for percentage activation time error at which they would be acceptable. The average single-iteration linear system solve time ratio (time for simulation)/(time for finest linear simulation) is given. Finest mesh *p* = 4 solution taken to be the “true” solution. Errors are worst-case over the three test nodes used. Full data in Table 4.

Maximum elt. diameter (cm)	Simulation degree	Worst nodal error reduction ratio	Time ratio	Example accuracy tolerance level (%)
0.0296	3	0.69	0.429	3
4	0.09	1.393	0.5

0.0148	1	2.80	0.176	12
2	0.38	0.495	2

0.0076	1	1 (by def.)	1 (by def.)	5
